# KDM5A, a H3K4me3 demethylase, regulates skin wound healing by promoting M2 macrophage polarization via suppression of Socs1

**DOI:** 10.3389/fphys.2025.1638356

**Published:** 2025-10-14

**Authors:** Jixun Zhang, Chao Wang, Xinxin Dong

**Affiliations:** Department of Plastic and Burn Surgery, The Second Hospital, Cheeloo College of Medicine, Shandong University, Jinan, China

**Keywords:** KDM5A, SOCS1, wound healing, fibroblasts, histone modification

## Abstract

**Introduction:**

The inflammatory phase is critical for successful wound healing, with macrophages playing a central role by polarizing into different functional phenotypes. KDM5A, a histone demethylase, can epigenetically suppress Socs1, a key negative regulator of immune responses. However, the specific roles and mechanisms of the KDM5A-Socs1 axis in macrophage polarization during cutaneous wound healing remain largely unknown. This study aims to elucidate the function of KDM5A in wound repair, focusing on its regulatory crosstalk with Socs1 in macrophages.

**Methods:**

We established a murine wound model to systematically evaluate wound closure kinetics, collagen deposition, healing scores, macrophage polarization dynamics, and inflammatory cytokine profiles. An *in vitro* co-culture system of macrophages and fibroblasts under KDM5A perturbation was used to assess its impact on fibroblast proliferation, migration, and angiogenic capacity. Mechanistic insights were gained through chromatin immunoprecipitation (ChIP) assays to determine the epigenetic regulation of Socs1 by KDM5A.

**Results:**

KDM5A expression was significantly downregulated in wound-associated macrophages and was inversely correlated with M2 polarization. Genetic ablation of KDM5A accelerated cutaneous wound closure, enhanced collagen deposition, and improved healing scores. Mechanistically, KDM5A deficiency elevated the activating histone marks H3K4me3 and H3K27ac at the Socs1 promoter, augmenting its transcriptional activation. The subsequent upregulation of Socs1 promoted M2 macrophage polarization, attenuated pro-inflammatory cytokine secretion, and stimulated fibroblast proliferation, migration, and angiogenesis.

**Discussion:**

Our findings demonstrate that KDM5A modulates wound healing by epigenetically regulating Socs1 expression. Downregulation of KDM5A in wound macrophages relieves the repression of Socs1, thereby driving M2 polarization and creating a pro-regenerative microenvironment that facilitates tissue repair. This study elucidates the KDM5A-Socs1 molecular axis as a key epigenetic regulator in wound healing and establishes a conceptual framework for developing novel therapeutic strategies.

## Introduction

Wound healing represents a sophisticated biological process that necessitates the precisely orchestrated integration of sequential phases - hemostasis, inflammation, proliferation, and remodeling (Kim et al., 2019; Kang et al., 2024). These distinct yet interdependent stages are fundamental to physiological tissue repair, facilitating progressive wound closure and functional restoration. However, when this carefully regulated cascade is disrupted, resulting in delayed or dysregulated healing progression, wounds may develop into persistent chronic lesions, presenting a substantial clinical burden that warrants urgent intervention (Wang et al., 2023; Chai et al., 2024).

Macrophages, ubiquitously distributed across mammalian tissues, play indispensable roles in tissue homeostasis maintenance and microenvironmental surveillance for infections and damage (Nobs and Kopf, 2021; Oza et al., 2024). These versatile immune cells exhibit two principal polarization states: classically activated pro-inflammatory (M1) and alternatively activated (M2) phenotypes. During the initial inflammatory phase of wound healing, circulating monocytes and resident dermal macrophages undergo activation and polarization into M1 macrophages, which are critical for phagocytic clearance of cellular debris and sustained secretion of pro-inflammatory mediators (Kang et al., 2024). The transition from inflammatory to proliferative phase is marked by a phenotypic switch from M1 to M2 macrophages, accompanied by progressive resolution of inflammation and initiation of tissue regeneration (Menzies et al., 2010; Daley et al., 2010). M2 macrophages orchestrate wound repair through coordinated regulation of collagen deposition, fibroblast proliferation and differentiation, as well as angiogenesis. In diabetic wounds, persistent M1 macrophage activation leads to sustained inflammatory responses that significantly impair healing progression (Ouyang et al., 2022). Conversely, functional impairment of M2 macrophages may contribute to the pathogenesis of chronic non-healing wounds. Therefore, precisely timed promotion of M2 macrophage polarization represents a crucial regulatory mechanism for ensuring physiological wound healing.

Although the association between macrophage polarization and wound healing is widely recognized, and the role of epigenetic regulation in determining cellular function is becoming increasingly clear, current research still faces two core challenges: First, the epigenetic regulatory mechanisms governing macrophage polarization during wound healing remain incompletely elucidated. While H3K4me3 modification is known as a key hallmark of transcriptional activation, its dynamic changes directly influence immune cell function. However, the role and mechanism of KDM5A—the enzyme responsible for H3K4me3 demethylation—in wound-associated macrophage polarization remain unreported. Consequently, its status as a key epigenetic regulator of the inflammation-repair balance in wounds remains unclear. Second, Socs1, a core molecule regulating macrophage homeostasis, exhibits unknown expression regulation mechanisms during wound healing. Existing studies confirm Socs1 suppresses excessive inflammatory responses and promotes M2 polarization. However, the factors regulating Socs1 expression in wound macrophages via epigenetic pathways, and how this regulation influences wound healing processes, remain inconclusive. These uncertainties limited the development of targeted wound treatment strategies based on macrophage polarization.

Suppressor of cytokine signaling 1 (Socs1), a pivotal member of the SOCS family, was initially identified within the secretory apparatus of IL-6-stimulated M1 mouse monocytic leukemia cells (Guo et al., 2024). This protein serves as a critical regulatory node for multiple cytokine signaling pathways involved in inflammatory responses and immune regulation (Liang et al., 2017). Beyond its well-characterized role as a negative feedback regulator of inflammation, SOCS1 functions as a molecular rheostat that governs macrophage homeostasis by modulating key signaling cascades essential for macrophage activation and functionality (Stoiber et al., 1999). Compelling evidence has established an inverse correlation between Socs1 expression levels and KDM5A activity (Zhao et al., 2020). KDM5A, a principal histone lysine demethylase, plays an indispensable role in epigenetic regulation. Histone modifications represent fundamental epigenetic mechanisms that control cellular processes, with specific patterns at gene regulatory elements serving as molecular signatures of chromatin accessibility (Feng et al., 2017). Through its enzymatic activity in removing trimethyl groups from histone H3 lysine 4 (H3K4me3), KDM5A dynamically modulates chromatin architecture, thereby controlling transcriptional initiation and subsequent gene expression profiles (Pavlenko et al., 2022; Kim et al., 2022; Hou et al., 2012).

KDM5A has been demonstrated to suppress astrocyte differentiation in neural progenitor cells (NPCs) through its catalytic removal of H3K4 methylation marks at the GFAP gene locus (Kong et al., 2018). Originally identified as an interacting partner of the retinoblastoma tumor suppressor protein (pRB), KDM5A exhibits pleiotropic functions across diverse cellular processes (Oser et al., 2019). Moreover, mutations in the homologous gene of KDM5A in *Drosophila* result in differentiation and cell growth defects (Gildea et al., 2000). Aberrant KDM5A expression has been implicated in the pathogenesis of various malignancies, neurological disorders, and metabolic diseases (Hou et al., 2012). However, the potential involvement of KDM5A in cutaneous wound repair and its mechanistic relationship with Socs1 in macrophage biology remain unexplored. Based on the aforementioned research gaps, this study aims to address three core scientific questions: 1) Characterize the expression changes of KDM5A during mouse skin wound healing and its effects on wound closure, collagen deposition, and angiogenesis; 2) Elucidate whether KDM5A participates in wound healing by regulating macrophage polarization; 3) Reveal whether KDM5A regulates Socs1 expression through epigenetic modifications, thereby mediating macrophage polarization and wound repair.

## Materials and methods

### Cell culture and transfection

The ANA-1 murine macrophage cell line (SUNNCELL, Wuhan), originally derived from C57BL/6 mice, and the normal human dermal fibroblast (NHDF) cell line (Pricella, Wuhan, CP-H103) were cultured in RPMI 1640 medium (Gibco, United States) supplemented with 10% fetal bovine serum (FBS, Gibco, United States), 100 μg/mL streptomycin, and 100 U/mL penicillin. Cells were maintained in an incubator (Thermo Fisher Scientific Inc., Waltham, MA, United States) at 37 °C with 5% CO_2_. When the cell density reached approximately 75%, transient transfection was performed using Lipofectamine 2000 (Invitrogen) with the following plasmids obtained from China Biotechnology Co., Ltd. (Beijing, China): overexpression (OE)-KDM5A, short hairpin RNA (sh)-KDM5A (sh1-KDM5A [5′-GGAACUGGGUCUCUUUUGA-3′], sh2-KDM5A [5′-GCAAAUGAGACAACGGAAA-3′]), sh-KDM5A+ shSocs1, and their corresponding controls (shNC, shKDM5A+ shNC, OE-NC). Following transfection, cells were cultured for an additional 48 h before collection.

Damaged ANA-1 cells were generated via lipopolysaccharide (LPS) treatment. ANA-1 cells were first seeded into 6-well plates and cultured in RPMI 1640 medium supplemented with 10% fetal bovine serum for 24 h until complete confluence. The medium was replaced with fresh medium containing 100 ng/mL LPS (Sigma-Aldrich). Cells were harvested at 0, 4, and 8 days post-treatment, and total RNA was extracted. KDM5A mRNA expression levels were detected via qRT-PCR to observe the dynamic changes in KDM5A under the inflammatory microenvironment.

### Skin wound healing model

Adult BALB/c mice were procured from Beijing HFK Bioscience Co., Ltd (China). Following established protocols (He et al., 2019), general anesthesia was induced through intraperitoneal administration of 1% pentobarbital sodium (200 mg/kg), after which the dorsal fur was aseptically removed. Full-thickness excisional wounds were surgically created by removing circular skin segments (diameter: 1.2 cm) from the dorsal region of each animal (n = 6 per experimental group, determined using G*Power 3.1 software). Concurrently, the mice were randomized to be in the shNC or shKDM5A groups, and each group received an injection of 2 × 10^6^ ANA-1 cells/mL, the injection was performed subcutaneously around the wound area from day 0 using a multiple-point injection technique to ensure even distribution of the cells around the wound margin. Every day, the wound area was observed, and at days 0, 4, and 8, the rate of wound healing was computed. The wound healing score was calculated based on three histological criteria: (1) degree of re-epithelialization, (2) granulation tissue formation, and (3) collagen deposition. Each criterion was scored on a scale from 0 to 3, with higher scores indicating better healing. The mice were euthanized at predetermined intervals, and skin samples were taken for additional examination. Sections of the wound samples were frozen and fixed in paraffin for use in histology and molecular investigations later on. In the final verification experiment, another set of mice with surgically induced skin defects (n = 6) were established and randomly assigned to the following groups: shNC group, shKDM5A group, shKDM5A + shNC group, and shKDM5A + shSocs1 group. Similar to the previous experiment, all groups were injected with 2 × 10^6^ cells/mL. The injection was performed around the wound margin via a multiple-point subcutaneous injection method on wound day 0.

### Analysis of skin wound healing

In this experiment, all general images were captured uniformly to ensure consistency and accuracy of the observations. Images were captured at three specific time points, namely, 0 days post-injury (dpi), 4 dpi, and 8 dpi, to closely monitor the progress of wound healing. When capturing images, a well-lit location was selected, and photos were taken from an angle perpendicular to the defective skin on the back of the mouse. In instances where new hair growth occurred on the intact skin of the mouse during the repair process, covering the damaged area, hair clippers were used to carefully trim the hair before capturing photos. This step ensures that the wound site is fully visible and not obscured by hair, enabling precise evaluation of wound healing. Subsequently, ImageJ was employed to measure the mouse skin wounds. Specifically, the area of dark-colored (red, red-black) parts in the images was uniformly counted as the unrepaired area.

### HE staining and healing statistics

Tissue sections were baked at 70 °C for 2 h to ensure proper fixation and adhesion to the slides. Perform dewaxing and hydration using a fully automatic staining machine, which typically takes approximately 45 min. Rinse the tissue sections with tap water for 15 min at a moderate flow rate to remove any residual wax or other impurities. Use HE staining kit (Beyotime, C0105S) to perform staining according to the instructions. Let the slides dry naturally and then mount them. Hematoxylin was used to stain the cell nuclei bright blue, the cartilage matrix and calcium salt particles dark blue, and the mucus gray-blue. Eosin was used to stain the cytoplasm in various tones of pink to peach. The eosinophilic granules within the cytoplasm exhibit vivid red coloration and potent reflecting qualities. Red blood cells were orange, and collagen fibers were a pale pink color. The color changes with the life cycle and pathological changes of the tissue or cell, as well as with the kind of tissue or cell.

### Masson staining

Utilize the Masson trichrome staining kit (Beyotime, C0189S) according to the manufacturer’s instructions to stain the tissue sections. After staining, quickly dehydrate the tissue sections in a series of ethanol solutions (70%, 80%, 90%, and absolute ethanol) for 10 s each to remove excess water and enhance staining intensity. Clear the tissue sections in xylene three times for 2 min each. Once the slides are dry, seal them with neutral resin, ensuring to avoid generating air bubbles that may interfere with microscopy. Collagen fibers, mucus, and cartilage should appear blue (or green if stained with light green solution), while the cytoplasm, muscle, cellulose, and glial cells should appear red. The nucleus should appear black or blue. Finally, observe and photograph the stained tissue sections under an optical microscope (Nikon, Tokyo, Japan) to visualize the staining patterns and structures. Utilize ImageJ software for further analysis quantification of collagen deposition.

### Immunohistochemistry (IHC)

Each group’s wound tissue slices were prepared for immunohistochemistry examination. First, alcohol gradients were used to dewaxe and dehydrate the paraffin sections. To stop endogenous peroxidase activity, the slices were then submerged in 3% methanol H2O2 for 20 min. The portions were submerged in a water bath containing a repair solution to retrieve the antigen. After antigen retrieval, the sections were dried for 20 min at room temperature before being blocked with regular goat serum blocking solution (C-0005, Haoran Bio, Shanghai, China). After that, the sections were incubated with primary antibody anti-CD31 (1:1,000; ab182981; Abcam, Cambridge, United Kingdom) for a whole night at 4 °C. Following the incubation of the primary antibody, the sections were treated for 1 hour at room temperature with a horseradish peroxidase-conjugated polymer anti-rabbit secondary antibody (1:1,000; Dako, Glostrup, Denmark). The sections were then stained with hematoxylin (PT001, Bogoo, Shanghai) for 1 minute after being treated with diaminoamphetamine (DAB; ST033, Whiga Biotechnology, Guangzhou, China) for visualization. After that, the sections underwent regular infiltration and mounting, were dehydrated with graded alcohol, and were blued with 1% ammonia. Ultimately, an optical microscope and a Nikon Eclipse 90i digital camera were used to see the slices. Using ImageJ software (NIH), immunohistochemical staining was examined, and the number of anti-CD31-positive vessels was used to calculate capillary density. To make sure the results were reliable, three parts from each sample were examined.

### Immunofluorescence (IF)

Analogous to immunofluorescence staining, ANA-1 cells were grown on coverslips as well as wound tissue slices. The primary antibody was incubated for a whole night at 4 °C, and the secondary antibody was incubated for an hour at room temperature, as per the standard procedures for immunofluorescence staining. DAPI staining solution (P0131; Beyotime, Shanghai, China) was used to see the nuclei of the cells. Anti-iNOS (abcam, ab178945) and Anti-CD163 (abcam, ab316218) were the primary antibodies employed in this investigation. The secondary antibody used for detection was an anti-rabbit antibody (1:1,000; 8889S; CST) coupled with Alexa Fluor^®^ 594. A confocal fluorescence microscope (Leica, Wetzlar, Germany) and the Leica LAS X system were used to image and capture sections and coverslips. For analysis, three sections from each sample were randomly selected and evaluated. Additionally, fluorescence intensity was quantitatively assessed using ImageJ software.

## ELISA

The ELISA kits provided by Beyotime (PT512, PI530, PT878, and PV957) were utilized to measure the levels of TNF-α, TGF-β1, and VEGF in both the wound skin tissues and ANA-1 cells of mice across various experimental groups. The assays were performed according to the instructions provided with each respective kit.

### Chromatin immunoprecipitation (ChIP) assay

ChIP detection was performed according to the method of Du using Magna ChIP A/G kit (Merck) (Du et al., 2020). ANA-1 cells were transfected with OE-NC, OE-KDM5A, si-NC, and si-KDM5A-1 for 48 h. The cells were fixed with 1% formaldehyde (Merck) at room temperature for 10 min, followed by the addition of glycine solution and cooling on ice for 5 min to stop the cross-linking reaction. After washing with PBS, the cell pellet was obtained and chromatin DNA was disrupted on ice. After centrifuged, the supernatant was mixed with ChIP buffer that contained protease inhibitors and a blocking solution was added. In order to decrease non-specific binding, the mixture was placed in a 4 °C incubation for 30 min. ChIP was performed by incubating the chromatin with 2 μg of KDM5A antibody (Abcam, China), 2 μg of H3K4me3 antibody (Abcam, ab8580), or 2 μg of H3K27ac antibody (Abcam, ab4729), along with 20 μL of Magna ChIP A/G magnetic beads, at 4 °C overnight. Following immunoprecipitation, the chromatin was eluted from the beads using ChIP elution buffer and RNase A mixture at 30 °C for 37 min, followed by proteinase K treatment at 62 °C for 2 h to reverse the cross-links. After purification of the DNA, the samples were subjected to qRT-PCR analysis to determine the enrichment of specific DNA sequences bound by the target proteins.

### Quantitative RT-PCR

Total RNA was extracted from wound skin tissues and ANA-1 cells of mice in different groups using TRIzol reagent (Invitrogen, Carlsbad, CA, United States). Oligo dT primers and PrimeScript^®^ RTase (Takara, Dalian, China) were used to reverse transcribe the isolated total RNA. Takara Holdings Inc. (Kyoto, Japan) was responsible for designing and synthesizing the primers of KDM5A (Table 1). Quantitative RT-PCR (qRT-PCR) was performed using SYBR®Premix Ex TaqTMII (Takara, Dalian, China) on a C1000TM Thermal Cycler. GAPDH served as the internal reference, and relative expression levels were calculated using the 2^(-△△Ct)^ method.

**TABLE 1:** Oligo dT primers of KDM5A and GAPDH used to reverse transcribe.

Gene	Sequence
GAPDH(Gene ID:14433,NM_001289726.1)	F: CGGCAAATTCAACGGCACAG
	R: GACATACTCAGCACCGGCCTCA
KDM5A (GeneID:214899,NM_145997.2)	F: AGCTGGCCCCTGAGTTATTT
	R: CCACAAACTCTCCAGCACAC

### Western blot

Total protein was extracted from mouse wound skin tissues and ANA-1 cells across various experimental groups utilizing radioimmunoprecipitation lysis buffer. The concentration of the extracted proteins was quantified using a BCA protein concentration assay kit (Beyotime, P0012). Following extraction, the proteins were separated via electrophoresis and subsequently transferred onto polyvinylidene fluoride membranes. The membranes were then incubated overnight with primary antibodies against KDM5A (1:10,000, Abcam, ab194286) and Socs1 (1:1,000, Abcam, ab280886). The subsequent day, the membranes were reprobed with a horseradish peroxidase-conjugated goat anti-rabbit IgG secondary antibody (1:1,000, Santa Cruz Biotechnology, Inc., Santa Cruz, CA, United States). Visualization of the membrane was performed, and the protein bands were quantified using the Bio-Rad ChemiDoc™ System in conjunction with ImageJ software. The relative protein expression was assessed by calculating the ratio of the gray value of the target band to that of Actin (1:10,000, rabbit, Santa Cruz Biotechnology, Inc., Santa Cruz, CA, United States).

### Tube formation assay

To prepare the 24-well plate for the tube formation assay, add 250 µL of basement membrane extract (BME, BD Biosciences, 354230) to each well, ensuring that the gel completely covers the bottom of the well. Incubate the plate for 30 min at 37 °C in an atmosphere containing 5% CO_2_ to facilitate the solidification of the BME. Next, 300 µL of culture medium was mixed with 10 µL of resuspended fibroblasts (approximately 3 × 10^4^ cells). Pre-coated Matrigel plates were carefully removed from the incubator, and 100 μL of cell suspension (approximately 10,000 cells) was gently and slowly added onto the surface of the solidified Matrigel in a 96-well plate. The plate was then carefully returned to a 37 °C, 5% CO_2_ incubator for culture, movement or shaking of the plate was avoided to prevent disruption of the forming tubular structures. HDFs were cultured in DMEM with 10% FBS and 1% penicillin–streptomycin. HUVECs (ATCC PCS-100-013) served as angiogenic cells. Growth factor-reduced Matrigel (Corning #354230) was added to 96-well plates (50 µL/well) and polymerized at 37 °C for 30 min. HUVECs (2 × 10^4^/well) were seeded on Matrigel either alone or co-cultured with HDFs (1 × 10^4^/well) by direct mixing (2:1) or by treatment with fibroblast-conditioned medium. Following the incubation period, employ a fluorescence microscope (Carl Zeiss, Oberkochen, Germany) to capture images of the cells in order to observe the formation of tubes. Subsequently, utilize ImageJ software to quantify the tube formation by assessing the average tube length along with other pertinent parameters.

### CCK-8 assay for cell proliferation

Cells were cultured in 96-well plates at a density of 3 × 10^3 cells per well. After allowing sufficient time for the cells to adhere completely, the culture medium was meticulously removed, and 100 μL of a 10-fold diluted CCK-8 reagent (Cell Counting Kit-8, C0038, Beyotime) was introduced to each well. The plates were subsequently incubated at 37 °C for a duration of 2 h. Upon completion of the incubation, the optical density (OD) of each well was assessed at a wavelength of 450 nm utilizing a microplate reader (SpectraMAX® 190, Molecular Devices, Sunnyvale, CA, United States). The relative OD value was determined by subtracting the OD value of the blank control from that of the experimental group. The blank group consisted of cell culture medium and CCK-8 solution without the addition of cells, serving as a baseline control for background absorbance.

### Transwell cell migration assay

Following the cultivation of fibroblasts to the logarithmic growth phase, the cells were subjected to digestion using 0.25% trypsin for a duration of 3 min and subsequently resuspended in serum-free Opti-MEMI medium (Invitrogen, United States). The cell density was adjusted to a range of 1–10 × 10^5 cells/mL. The resulting cell suspension was then introduced into the upper chambers of 8 μm Transwell inserts (Corning, NY, United States) at a volume of 100 μL per well, utilizing a total of three wells. Concurrently, 600 μL of culture medium supplemented with 10% RPMI-1640 was added to the lower chamber of a 24-well plate to facilitate incubation. The plate was subsequently placed in an incubator for a period of 12 h to promote cell migration. After incubation, the culture medium was aspirated from the upper chambers, and the cells that had migrated to the underside of the membrane were fixed with 600 μL of 4% paraformaldehyde for 30 min. The fixed cells were then stained with 800 μL of a 0.2% crystal violet dye solution for 15 min. After the staining process, any excess dye solution was discarded, and the membranes were gently washed with phosphate-buffered saline (PBS) to eliminate non-specific staining. Finally, the number of stained cells was quantified by counting in five randomly selected fields using an inverted microscope (Leica DMi8-M, Co. Ltd, Solms, Germany), and the average value was calculated to assess the migratory capacity of the fibroblasts.

### Statistical analysis

The means ± standard error of the mean (SEM) is used for statistical analysis of between-group mean comparisons, while the means ± standard deviation (SD), indicating the variability of the raw data, the relevant values have been annotated in the results and figure captions. All continuous data were tested for normality using the Shapiro-Wilk test. Given the normal distribution, we used unpaired two-tailed Student’s t-tests for comparisons between two groups and one-way ANOVA for multiple groups. Bonferroni correction was applied post-ANOVA to adjust for Type I errors when comparing >2 groups. All statistical analyses were performed utilizing GraphPad Prism 5.0 (GraphPad Software, La Jolla, CA, United States). A significance level of P < 0.05 was established for all tests.

## Result

### KDM5A is expressed at low levels in wound healing-related macrophages

**FIGURE 1:**
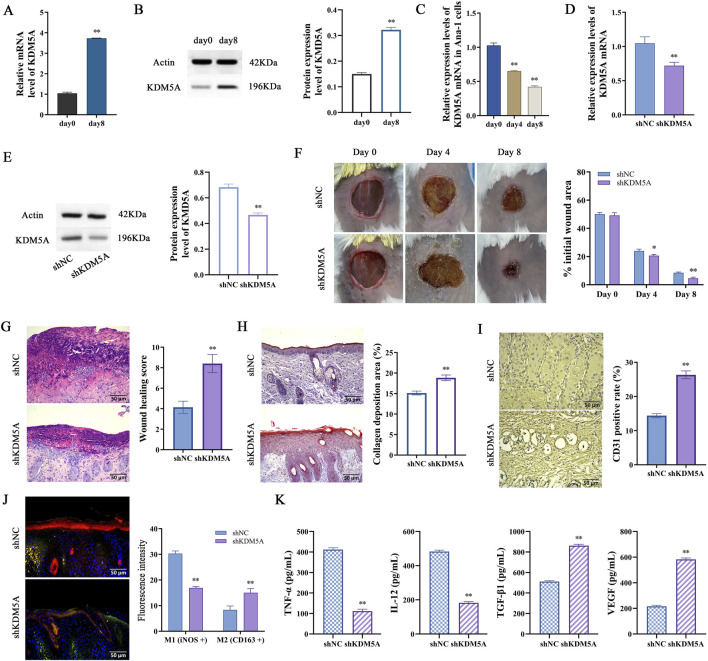
Molecular and histological characterization of KDM5A in wound healing. **(A,B)** Temporal profiling of KDM5A transcriptional and translational expression in murine dorsal wound tissues (n = 6). Data were tested for normality using Shapiro-Wilk test, and comparisons between day 0 and day 8 were performed using unpaired two-tailed Student’s t-test. P < 0.01 vs. day 0.; **(C)** Dynamic changes in KDM5A mRNA levels in LPS-treated ANA-1 cells (n = 3). Data were tested for normality using Shapiro-Wilk test, and comparisons among time points were performed using one-way ANOVA followed by Bonferroni post-hoc test. P < 0.01 vs. day 0.; **(D–K)** Comprehensive evaluation of shKDM5A-transfected cutaneous wound repair: **(D,E)** qRT-PCR and WB validation of KDM5A knockdown efficiency on day 5 post-wounding; **(F)** Quantitative assessment of wound contraction (n = 6, means ± SD). Data were tested for normality using Shapiro-Wilk test; comparisons between shNC and shKDM5A at each time point were performed using unpaired two-tailed Student’s t-test. *P < 0.05, P < 0.01 vs. shNC.; **(G)** Histopathological examination by hematoxylin-eosin staining; **(H)** Connective tissue matrix analysis via Masson’s trichrome staining (n = 6, means ± SD); **(I)** Neovascularization assessment through CD31 immunohistochemistry at day 10 post-wounding; **(J)** Macrophage polarization status determined by iNOS (red)/CD163 (green) immunofluorescence co-staining with DAPI nuclear counterstain (blue); **(K)** Cytokine microenvironment profiling measuring TNF-α, IL-12, TGF-β1 and VEGF concentrations. Panels **(G–K)** show data from 10 days after wounding, data were tested for normality using Shapiro-Wilk test, and comparisons between shNC and shKDM5A were performed using unpaired two-tailed Student’s t-test. P < 0.01 vs. shNC.

Following induction of dorsal cutaneous wounds in mice, quantitative analysis revealed a significant upregulation of both KDM5A gene and protein expression levels at day 8 post-injury (P < 0.01; Figures 1A,B), suggesting its potential functional involvement in the wound repair process. In striking contrast, ANA-1 cells exhibited a progressive downregulation of KDM5A mRNA expression at the 4- and 8-day post-injury time points (P < 0.01; Figure 1C), demonstrating active transcriptional suppression of KDM5A during wound healing progression.

### Knockdown of KDM5A promotes skin wound healing in mice

In this investigation, we employed a well-established murine dorsal skin wound model to systematically evaluate the functional consequences of KDM5A suppression on wound repair processes. Successful KDM5A knockdown was quantitatively verified through qRT-PCR and WB (Figures 1D,E). Comparative assessment of wound closure kinetics between shNC and shKDM5A groups revealed distinct temporal patterns. At the early repair phase (4 dpi), the shKDM5A cohort exhibited a modest but statistically significant reduction in wound closure relative to controls (20.64% ± 0.95% vs. 23.97% ± 1.24%, P < 0.05). However, by 8 dpi, KDM5A-deficient mice demonstrated markedly enhanced wound resolution, with residual wound areas measuring 4.57% ± 0.81% compared to 8.51% ± 0.55% in controls (P < 0.01; Figure 1F). Histomorphometric analysis of HE-stained sections confirmed substantial neodermal formation and adipocyte recruitment in shKDM5A specimens, accompanied by significantly improved wound healing scores (P < 0.01; Figure 1G).

The critical role of collagen matrix deposition in tissue regeneration was evidenced by Masson’s trichrome staining, which revealed significantly increased collagen content in shKDM5A wounds (15.13% ± 0.48% vs. control, P < 0.01; Figure 1H). Furthermore, immunohistochemical quantification demonstrated enhanced CD31^+^ microvascular density in KDM5A-deficient tissue (P < 0.01; Figure 1I), suggesting improved angiogenic potential. Immunofluorescence profiling identified a distinct macrophage polarization shift in shKDM5A wounds, characterized by reduced iNOS signal intensity concomitant with elevated CD163 expression (P < 0.01; Figure 1J), indicative of preferential M2 macrophage activation. Cytokine analysis revealed significant downregulation of pro-inflammatory mediators (TNF-α, IL-12) with concomitant upregulation of reparative factors (TGF-β1, VEGF) in the shKDM5A group (Figure 1K). These collective findings establish KDM5A as a critical epigenetic regulator of cutaneous wound repair through modulation of macrophage polarization and extracellular matrix remodeling.

### KDM5A modulates macrophage polarization and inflammatory responses

**FIGURE 2:**
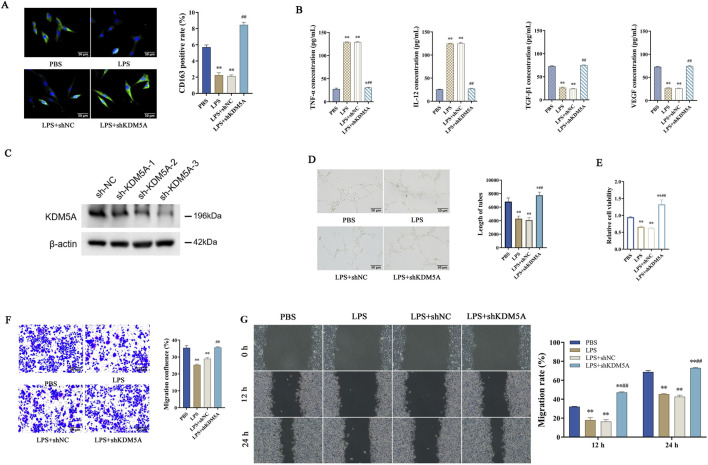
**(A,B)** Establishment of ANA-1 cell model stably expressing shKDM5A after transfection; **(A)** Immunofluorescence detection of CD163 fluorescence signal intensity in cells; **(B)** ELISA detects the expression levels of TNF-α, IL-12, TGF-β1 and VEGF in cells. Data were tested for normality using Shapiro-Wilk test, and comparisons among groups were performed using one-way ANOVA followed by Bonferroni post-hoc test. P < 0.01 vs. PBS; ##P < 0.01 vs. LPS + shNC. **(C–F)** Establishment of NHDF cell model stably expressing shKDM5A after transfection; **(C)** KDM5A expression in NHDF cells transfected with KDM5A-targeting shRNAs. **(D)** Analysis of tube formation capacity in human dermal fibroblasts (NHDFs) co-cultured with shRNA-treated ANA-1 macrophages; **(E)** CCK-8 to detect cell proliferation; **(F)** Transwell migration assay to detect cell migration density; **(G)** Cell migration rates at 12 h and 24 h after cell treatment. Data were tested for normality using Shapiro-Wilk test, and comparisons among groups were performed using one-way ANOVA followed by Bonferroni post-hoc test. P < 0.01 vs. shNC/LPS + shNC; ##P < 0.01 vs. LPS. *represents *P* < 0.05, **P < 0.01, ^#^ *P* < 0.05, ^##^ *P* < 0.01.

Immunofluorescence analysis of ANA-1 cells demonstrated markedly attenuated CD163 signal intensity in both LPS-treated and LPS + shNC control groups relative to PBS controls. In contrast, LPS-stimulated cells with KDM5A knockdown exhibited significantly enhanced CD163 fluorescence (P < 0.01; Figure 2A), indicating that KDM5A suppression promotes M2 macrophage polarization-a finding consistent with the elevated CD163 expression observed in murine dermal wound tissues (Figure 1J). Quantitative cytokine profiling revealed that while LPS challenge robustly induced pro-inflammatory responses, shKDM5A transfection substantially mitigated these effects. Specifically, LPS exposure significantly increased TNF-α and IL-12 secretion while reducing TGF-β1 and VEGF production compared to PBS controls (P < 0.01). Notably, KDM5A-depleted cells showed significant reversal of these cytokine alterations when compared to LPS + shNC counterparts (P < 0.01; Figure 2B). These collective findings demonstrate that KDM5A silencing attenuates inflammatory cascades while fostering an anti-inflammatory microenvironment conducive to cellular repair processes.

### Silencing KDM5A promotes fibroblast vascularization, proliferation, migration and invasion

Fibroblasts exhibit functional parallels with vascular endothelial cells in their capacity to support neovascularization. We successfully knocked down KDM5A in NHDFs using distinct shRNAs targeting KDM5A (Figure 2C). *In vitro* angiogenesis assays demonstrated that LPS treatment significantly impaired tubular network formation, whereas KDM5A knockdown in NHDF cells markedly augmented their angiogenic potential, as reflected by increased vascular length (P < 0.01; Figure 2D). Subsequent CCK-8 proliferation analysis revealed that while LPS exposure substantially attenuated NHDF cell growth, shKDM5A transfection significantly enhanced cellular proliferative capacity (P < 0.01; Figure 2E). Transwell invasion assays further established that KDM5A suppression promoted NHDF cell invasiveness (P < 0.01; Figure 2F). Consistent with these observations, temporal migration analysis demonstrated progressive enhancement of NHDF cell motility at both 12 h and 24 h post-KDM5A silencing (P < 0.01; Figure 2G). These data collectively indicate that KDM5A ablation potentiates fibroblast-mediated vascularization while concomitantly augmenting their proliferative, migratory and invasive properties.

### KDM5A epigenetically represses Socs1 expression via H3K4me3 demethylation

**FIGURE 3:**
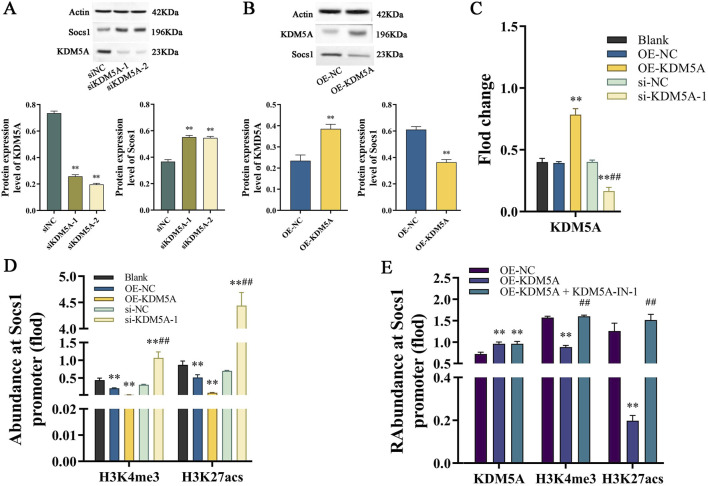
**(A)** WB detects KDM5A and Socs1 protein expression in ANA-1 cells after siKDM5A-1 and siKDM5A-2 treatment; **(B)** WB detects KDM5A and Socs1 protein expression in ANA-1 cells after OE-KDM5A treatment; **(C)** PCR detects KDM5A mRNA expression levels in ANA-1 cells in different treatment groups. Data were tested for normality using Shapiro-Wilk test; comparisons between control (si-NC/OE-NC) and treatment (si-KDM5A/OE-KDM5A) groups were performed using unpaired two-tailed Student’s t-test. *P < 0.05, P < 0.01 vs. si-NC/OE-NC. **(D,E)** ChIP-seq detects the enrichment of two histone modifications, H3K4me3 and H3K27ac, on the Socs1 promoter in ANA-1 cells in different treatment groups. Data were tested for normality using Shapiro-Wilk test, and comparisons among groups were performed using one-way ANOVA followed by Bonferroni post-hoc test. P < 0.01 vs. si-NC/OE-NC; ##P < 0.01 vs. OE-KDM5A.

Initial validation of KDM5A modulation efficacy was performed through comprehensive assessment of mRNA and protein expression profiles in siKDM5A-1, siKDM5A-2, and OE-KDM5A groups using WB and quantitative PCR analyses. Notably, KDM5A knockdown consistently upregulated Socs1 protein expression, establishing its regulatory role in Socs1 expression control (Figures 3A–C). Chromatin immunoprecipitation assays subsequently revealed KDM5A’s direct binding to the Socs1 promoter region, with KDM5A overexpression significantly reducing H3K4me3 and H3K27ac histone mark deposition, while KDM5A depletion conversely enhanced these activating modifications (Figure 3D), demonstrating KDM5A’s function as a negative regulator of Socs1-associated histone modifications.

Pharmacological inhibition experiments further corroborated these findings. Compared to OE-NC controls, KDM5A-overexpressing ANA-1 cells exhibited diminished H3K4me3 and H3K27ac enrichment at the Socs1 promoter concomitant with increased KDM5A occupancy (P < 0.01; Figure 3E). Importantly, KDM5A inhibitor treatment effectively rescued the histone modification profile, restoring H3K4me3 and H3K27ac levels (P < 0.01). These data collectively establish that KDM5A-mediated recruitment to the Socs1 promoter orchestrates epigenetic silencing through erasure of activating histone marks, thereby suppressing Socs1 transcriptional activation in ANA-1 cells.

### KDM5A regulates Socs1 to affect M2 polarization of macrophages and vascularization and migration of fibroblasts

**FIGURE 4:**
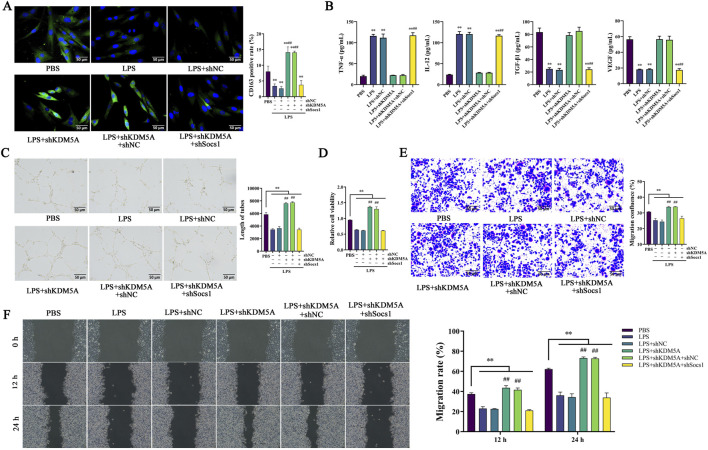
**(A,B)** Establishment of ANA-1 cell model stably expressing shKDM5A and shKDM5A + shSocs1 after transfection; **(A)** Immunofluorescence detection of CD163 fluorescence signal intensity in cells; **(B)** ELISA detects the expression levels of TNF-α, IL-12, TGF-β1 and VEGF in cells; **(C–F)** Establishment of NHDF cell model stably expressing shKDM5A and shKDM5A + shSocs1 after transfection; **(C)** Analysis of blood vessel formation in cells; **(D)** CCK-8 to detect cell proliferation; **(E)** Transwell migration assay to detect cell migration density; **(F)** Cell migration rates at 12 h and 24 h after cell treatment; Data were tested for normality using Shapiro-Wilk test, and comparisons among groups were performed using one-way ANOVA followed by Bonferroni post-hoc test. *represents *P* < 0.05, **P < 0.01, ^#^ *P* < 0.05 and ^##^ *P* < 0.01.

We found that the regulatory effect of KDM5A is mainly achieved by affecting the expression of Socs1. Therefore, based on stably expressing shKDM5A, the Socs1 gene was silenced to verify the regulatory effect of KDM5A on Socs1 in macrophages and fibroblasts. Compared to the LPS + shKDM5A + shNC group, the LPS + shKDM5A + shSocs1 group led to a significant decrease in CD163 fluorescence in ANA-1 cells (*P* < 0.01), promoting polarization of macrophages towards the M1 phenotype (Figure 4A). This led to elevated concentrations of the inflammatory cytokines TNF-α and IL-12, while concurrently resulting in reduced levels of the growth factors TGF-β1 and VEGF (Figure 4B). This further aggravates the inflammatory response. Concurrently, the inhibition of KDM5A and Socs1 led to a marked decrease in the capabilities of NHDF cells regarding blood vessel formation, as well as cell proliferation, migration, and invasion (P < 0.01) (Figures 4C–F). These findings indicate that the KDM5A-Socs1 pathway is crucial in modulating immune responses and angiogenesis.

### KDM5A regulates wound healing in mice by regulating the expression of Socs1

**FIGURE 5:**
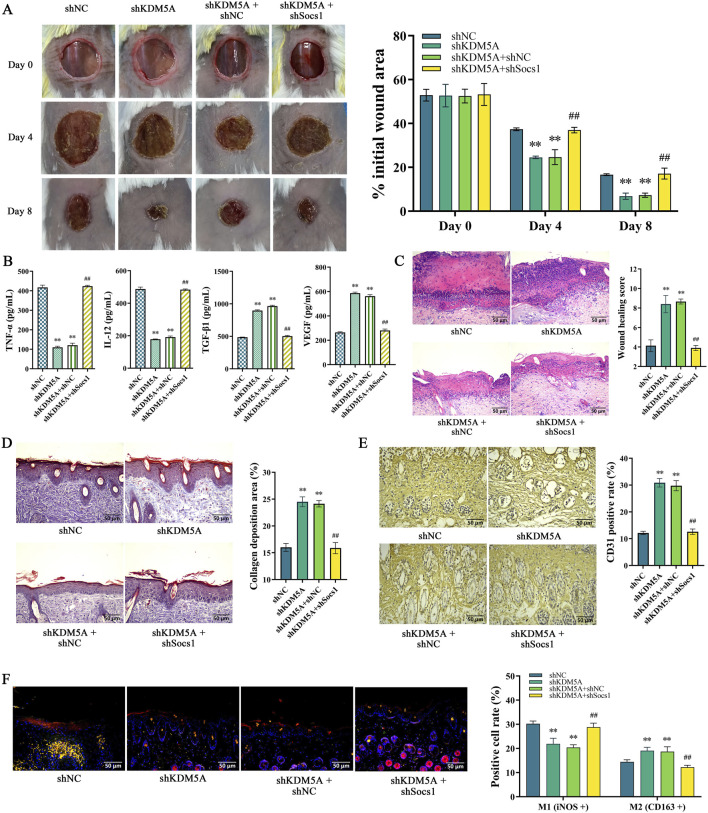
**(A–F)** Establishment of mouse back skin injury model stably expressing shKDM5A and shKDM5A + shSocs1 after transfection; **(A)** Analysis of the wound healing area of mouse back skin; **(B)** ELISA detects the expression levels of TNF-α, IL-12, TGF-β1 and VEGF in the mouse back skin wound tissue; **(C)** HE staining results of mouse back skin wound tissue; **(D)** Masson trichrome staining experimental results of mouse back skin wound tissue; **(E)** Immunohistochemical detection of CD31 expression in mouse back skin wound tissue; **(F)** Immunofluorescence detects the signal intensity of iNOS and CD163 in the mouse back skin wound tissue; Data were tested for normality using Shapiro-Wilk test, and comparisons among groups were performed using one-way ANOVA followed by Bonferroni post-hoc test. *represents *P* < 0.05, **P < 0.01, ^#^ *P* < 0.05 and ^##^ *P* < 0.01.

It was verified in the back skin injury model of mice stably expressing shKDM5A and shKDM5A + shSocs1. Statistics on the wound repair area of mice at 4 dpi and 8 dpi showed that both the shKDM5A group and the shKDM5A + shNC group were lower than the shNC group (*P* < 0.01). Compared with the shKDM5A + shNC group, the repair rate of the shKDM5A + shSocs1 group increased (*P* < 0.01; Figure 5A). It was also found that the wound healing score, collagen deposition area, and the proportion of CD31 in skin tissue were significantly lower in the shKDM5A + shSocs1 group than in the shKDM5A + shNC group (*P* < 0.01; Figures 5C–E). This suggests that KDM5A may have a specific function in the regulation of Socs1. When KDM5A is silenced, wound repair is accelerated, while silencing Socs1 reverses this accelerated effect and affects the wound repair process.

Immunofluorescence analysis of wound tissues revealed that combined KDM5A/Socs1 silencing significantly reduced CD163 signal intensity (P < 0.01; Figure 5F), indicative of M1-polarized macrophage predominance. This phenotypic shift correlated with elevated pro-inflammatory mediators (TNF-α, IL-12) and iNOS expression (P < 0.01; Figures 5B,F), while concurrently suppressing reparative factors (TGF-β1, VEGF; P < 0.01; Figure 5B). Although M1 macrophages facilitate early wound debridement, their sustained activation promotes tissue damage and chronic inflammation. These findings collectively demonstrate that KDM5A-mediated wound healing acceleration is mechanistically dependent on Socs1 expression, with Socs1 ablation reinstating inflammatory dominance and impairing tissue regeneration.

## Discussion

The inflammatory response constitutes a fundamental determinant of cutaneous wound repair, with studies demonstrating markedly impaired tissue regeneration following macrophage depletion *in vivo* (Kirwin et al., 2024; Lucas et al., 2010). The remarkable phenotypic plasticity of macrophages is profoundly influenced by dynamic microenvironmental cues (Amit et al., 2016; Thai et al., 2023), with the temporal transition from pro-inflammatory M1 to reparative M2 polarization representing a critical determinant of successful tissue regeneration (Hu et al., 2024). This transition typically occurred after the initial stages of the inflammatory response and peaked during the process of wound healing (Mao et al., 2021). Notably, between days 1 and 3, approximately 85% of macrophages exhibited the M1 phenotype, whereas during days 5–7, the proportion of M1 macrophages decreased to 80%–85%, concomitantly with an increase in the proportion of M2 macrophages (Miao et al., 2012). The delicate balance between M1 and M2 macrophage populations serves as a key regulator of inflammatory modulation and tissue repair processes (Mesquida-Veny et al., 2021), with the molecular mechanisms governing polarization - encompassing diverse signaling cascades, transcriptional regulators, and epigenetic modifications - having emerged as a focus of intensive investigation (Sica and Mantovani, 2012; Zou et al., 2024).

Among these regulatory mechanisms, the H3K4me3-specific demethylase KDM5A has been identified as a pivotal epigenetic modulator of chromatin architecture and transcriptional programs. As a versatile epigenetic regulator, KDM5A orchestrates multiple cellular processes including differentiation, proliferation and apoptotic regulation. Our findings establish that KDM5A-mediated H3K4me3 demethylation modulates M2 macrophage polarization through targeted suppression of Socs1 expression, thereby influencing cutaneous wound repair outcomes. These findings not only deepen our understanding of the molecular mechanisms underlying skin wound healing but also provide new therapeutic strategies and potential drug targets for enhancing tissue repair and managing chronic wounds.

Accumulating evidence indicates that KDM5A plays a crucial role in various cellular processes, including DNA break repair, cell cycle progression, cellular senescence, NK cell activation, mitochondrial function, and circadian rhythm (Zhao et al., 2020; Price, 2017; Guo et al., 2019). Notably, KDM5A maintains neural progenitor cell pluripotency by suppressing astrocytic differentiation through epigenetic silencing of lineage-specific genes (Kong et al., 2018). Our findings indicate that KDM5A is expressed at reduced levels in macrophages associated with wound healing and exhibits a negative correlation with the M2 polarization of these macrophages. The M2 macrophage phenotype, characterized by signature markers including CD163, CD206, IL-10, and ARG-1, orchestrates type 2 immune responses while promoting tissue remodeling through anti-inflammatory cytokine production, pro-angiogenic factor secretion, and extracellular matrix deposition (Pan et al., 2020). The shift from a wound environment primarily characterized by pro-inflammatory macrophages to one that is anti-inflammatory during the intermediate and later stages is crucial for reducing inflammation and facilitating effective wound healing. (Zhou et al., 2024).

Notably, while KDM5A silencing accelerates wound healing by promoting M2 polarization, the timing of this shift requires careful consideration. The early inflammatory phase, dominated by M1 macrophages, is critical for pathogen clearance and debris removal. Our data show that KDM5A knockdown reduces but does not eliminate pro-inflammatory cytokines (TNF-α, IL-12; Figures 1K, 2B), suggesting a modulated rather than abrogated inflammatory response. However, in non-sterile clinical settings, prematurely enhancing M2 polarization could theoretically increase infection risk by blunting the initial immune defense. Future studies should explore context-specific strategies, such as timed KDM5A inhibition post-injury, to balance inflammation and repair, ensuring both efficient healing and host protection against pathogens.

Socs1 functions as a critical negative regulator of JAK-STAT signaling pathways, limiting excessive inflammatory responses through feedback inhibition of STAT3 activation (Ila et al., 2024; Liu et al., 2021). The development of Socs1 mimetic peptides, such as Tkip which replicates the JAK-interacting domain of Socs1, has shown therapeutic potential in inflammatory disorders through selective inhibition of dysregulated immune responses in myeloid and lymphoid lineages (Ahmed et al., 2018; Sharma and Larkin, 2019).

Histone methylation represents a dynamically regulated epigenetic modification governed by lysine-specific demethylases (KDMs) (Pedersen and Helin, 2010). Recognizing the pivotal role of epigenetic regulation in both physiological and pathological contexts, we performed chromatin immunoprecipitation analyses that demonstrated KDM5A deficiency induces pronounced enrichment of both H3K4me3 and H3K27ac modifications. The H3K4me3 mark serves not merely as a hallmark of transcriptionally active promoters, but also identifies genomic loci poised for transcriptional activation (AJ et al., 2007). H3K27ac typically associates with enhancers and facilitates gene expression. Additionally, KDM5A-mediated demethylation of H3K4me3 has been implicated in the etiology of osteoporosis (Wang et al., 2016). In quiescent natural killer cells, KDM5A occupancy at the Socs1 promoter region results in diminished H3K4me3 deposition and concomitant chromatin remodeling (Zhao et al., 2020). These regulatory elements—enhancers that amplify transcriptional output of distal genes and promoters that initiate proximal gene expression—collectively establish a permissive chromatin state when marked by elevated H3K4me3 and H3K27ac levels, thereby facilitating Socs1 promoter accessibility.

During the initial inflammatory phase of wound healing, M1 macrophages secrete a variety of proteins into the wound, including pro-inflammatory cytokines like TNF-α, IL-12, IL-6, and iNOS (Mogami, 2022). However, our examination of changes in iNOS and CD163 fluorescence signals at skin wounds in a murine skin injury model suggests that silencing KDM5A may facilitate the polarization of macrophages towards the M2 phenotype while reducing the secretion of pro-inflammatory mediators. This observation was also corroborated in shKDM5A-treated macrophages (ANA-1 cells), where shKDM5A reversed the lipopolysaccharide (LPS)-induced inflammatory response. The enhanced fluorescence signal of CD163 further emphasizes the polarization of macrophages towards the M2 phenotype. These results highlight the important function of KDM5A in modulating macrophage polarization and facilitating skin wound healing. Additionally, Socs1 is critical in the regulation of Toll-like receptor 4 (TLR4) signaling in response to bacterial lipopolysaccharide (LPS). Consequently, mice lacking Socs1 demonstrate an increased production of inflammatory cytokines by both macrophages and dendritic cells (Stoiber et al., 1999; Mansell et al., 2006; Whyte et al., 2011). Notably, KDM5A modulates the expression of Socs1, thereby influencing wound healing, as evidenced by observations in the group where KDM5A and Socs1 were simultaneously silenced.

In the proliferative phase of wound healing, fibroblasts migrate to the dermal layer and commence the synthesis of immature extracellular matrix (ECM) proteins, including EDA, brunectin, and type III collagen, as well as growth factors such as TGF-β1 (Bielefeld et al., 2013). The recruitment of fibroblasts to the wound site is an essential mechanism for the formation of granulation tissue as well as for the synthesis and deposition of collagen (Diller and Tabor, 2022). Through conditioned media experiments employing shKDM5A- and shSocs1-treated macrophage supernatants (Lei et al., 2023). Consequently, the loss of KDM5A was found to promote fibroblast angiogenesis, proliferation, migration, and invasion, while silencing Socs1 exerted an inhibitory effect. These findings further underscore the regulatory relationship between KDM5A and Socs1.

Extensive research has established that augmenting M2 macrophage polarization substantially enhances wound repair kinetics by orchestrating cellular proliferation, migratory responses, neovascularization, and the sequential progression of healing cascades (Louiselle et al., 2021; Chen et al., 2024). M2 macrophages possess the ability to stimulate the activation of vascular endothelial cells and fibroblasts through the release of vascular growth factors, including TGF-β and VEGF (Zhang et al., 2021; Mantovani et al., 2013). While physiologically essential for tissue homeostasis, TGFβ assumes particular significance in pathological contexts by modulating inflammatory responses and coordinating wound repair processes (Tie et al., 2022; On et al., 2021; Weng et al., 2020). TGFβ1 has been demonstrated to suppress the expression of CCL3, a chemokine implicated in the recruitment of inflammatory cells, via the ERK signaling pathway (Zhang et al., 2017). Furthermore, Vascular Endothelial Growth Factor (VEGF) is integral to the processes of tissue repair and regeneration, as it aids in the reconstruction of the vascular network and enhances the supply of oxygen and nutrients (Cheng et al., 2024a). Our experimental data corroborate the indispensable contributions of both TGF-β1 and VEGF to wound closure efficacy. Importantly, Socs1 emerges as a master regulator of M2 polarization (Liu et al., 2008; Arnold et al., 2014), with its expression being markedly upregulated in M2 macrophages across experimental systems (McCormick and Heller, 2015; Duncan et al., 2017).

Neovascularization constitutes a fundamental requirement for successful wound repair, as inadequate vascular perfusion compromises nutrient delivery and impedes regenerative processes (Huang et al., 2024). The endothelial-specific marker CD31 (PECAM-1) (He et al., 2024), whose expression was significantly elevated following KDM5A knockdown in our murine wound model, mediates intercellular adhesion to maintain vascular integrity and functionality. The observed phenotypic alterations may stem from KDM5A-mediated transcriptional activation of Socs1, a critical inflammatory modulator. While the initial inflammatory phase is indispensable for microbial clearance and necrotic tissue removal (Poddar et al., 2024), macrophages play pivotal roles in wound debridement through phagocytic elimination of pathogens, cellular debris, and inflammatory mediators (Koudstaal et al., 2024). The polarization of macrophages influences the corresponding alterations in cytokine profiles and associated markers (Orecchioni et al., 2019; Zhao et al., 2023). Consequently, the Enzyme-Linked Immunosorbent Assay (ELISA) is utilized to conduct a more detailed evaluation of the expression levels of critical factors, including TNF-α and IL-12. The reduction in TNF-α and IL-12 levels suggests that the decrease in KDM5A and the increase in Socs1 may mitigate the extent of the inflammatory response. Consistent with *in vitro* studies, silencing of KDM5A led to elevated levels of TGF-β1 and VEGF and reduced levels of TNF-α and IL-12 in ANA-1 cells.

In our cutaneous injury model, KDM5A depletion accelerated wound closure and enhanced collagen matrix deposition. As the predominant structural component of connective tissue, collagen establishes a provisional extracellular matrix that maintains wound architecture and integrity (Cheng et al., 2024b). Presently, topical application of exosomes has been shown to augment collagen deposition, expedite wound healing, and enhance overall cosmetic outcomes (Ash et al., 2024). Furthermore, the bioactive groups present on the surface of collagen molecules can interact with cell surface receptors, thereby initiating cell signaling pathways (Wei et al., 2024). Histopathological evaluation of HE-stained sections revealed superior healing indices in shKDM5A-treated wounds, whereas dual KDM5A/Socs1 knockdown phenocopied control groups. This observation aligns with established mechanisms whereby Socs1 overexpression attenuates inflammation by limiting inflammatory cell infiltration (Yu et al., 2011), while its deficiency activates JAK1/STAT1 signaling to promote M1 polarization (Liang et al., 2017). Our findings collectively position KDM5A as a critical epigenetic modulator that fine-tunes macrophage polarization through Socs1 regulation, thereby optimizing the inflammatory-reparative balance during cutaneous wound healing.

A potential limitation of the present study lies in the use of BALB/c mice as the *in vivo* model while employing ANA-1 macrophages derived from C57BL/6 mice. This strain mismatch raises theoretical concerns regarding immune compatibility, as allogeneic cell transfer between distinct mouse strains may trigger immune recognition and clearance, potentially confounding the interpretation of paracrine effects on wound healing.

In our experimental design, we sought to mitigate this risk by restricting ANA-1 cell administration to a single local injection at day 0, focusing on the acute wound healing phase (≤8 days) during which immune surveillance of foreign cells is relatively attenuated. Consistent with this approach, we observed no overt signs of accelerated cell clearance or aberrant inflammatory infiltration, suggesting that the short-term paracrine effects of ANA-1 cells, such as cytokine secretion, modulation of macrophage polarization, were not significantly disrupted by immune rejection. However, we acknowledge that this does not entirely eliminate the possibility of subclinical immune responses, which could subtly alter the kinetics of macrophage-mediated repair or the magnitude of downstream effects on fibroblasts and angiogenesis. Future studies would benefit from validating these findings in a syngeneic model, which would eliminate strain-related immune variables and strengthen the generalizability of the KDM5A-Socs1 axis in regulating cutaneous wound healing. Such a model would provide more definitive evidence for the therapeutic potential of targeting KDM5A in macrophage-driven tissue repair.

## Conclusion

In conclusion, our investigation has demonstrated that genetic ablation of KDM5A significantly accelerates cutaneous wound repair in murine models, as evidenced by enhanced collagen matrix deposition, improved histological healing indices, modulated macrophage polarization dynamics, and optimized inflammatory mediator profiles. Mechanistically, KDM5A depletion potentiates fibroblast functionality by augmenting proliferative capacity, migratory potential, invasive properties, and pro-angiogenic activity. These phenotypic alterations are mediated through epigenetic remodeling characterized by increased H3K4me3 and H3K27ac histone modifications at the Socs1 locus, resulting in transcriptional activation of Socs1. The consequent upregulation of Socs1 orchestrates macrophage repolarization towards the pro-reparative M2 phenotype, attenuates pro-inflammatory cytokine secretion, and ultimately facilitates an optimized microenvironment for efficient cutaneous regeneration.

## Limitations

1. Allogeneic mismatch between mouse strain and cell line. A key methodological limitation is the use of BALB/c mice with ANA-1 macrophages derived from C57BL/6 mice. To our knowledge, this specific allogeneic combination has no prior literature support, raising concerns about immune compatibility. Although we mitigated this risk by restricting ANA-1 cells to a single subcutaneous injection (day 0) and focusing on the acute healing phase (≤8 days)—and observed no overt immune rejection—subclinical immune effects cannot be fully ruled out. Future studies should validate these findings in a syngeneic model to eliminate strain-related immune interference.
2. Lack of in-depth analysis of the temporal regulation of macrophage polarization. Wound healing is a dynamic process, with M1 macrophages responsible for clearing pathogens and necrotic tissue in the early stages (1–3 days) and M2 macrophages dominating repair in the middle stages (5–7 days). The temporal balance between the two is critical for healing. While studies have observed that KDM5A knockdown promotes M2 polarization, they have not systematically analyzed the dynamic regulatory effects of KDM5A on macrophage polarization at different stages of wound healing (0 days, 4 days, 8 days).

## Data Availability

The original contributions presented in the study are included in the article/Supplementary Material, further inquiries can be directed to the corresponding author.
