# Supplementary figures and images for: KDM5A, a H3K4me3 demethylase, regulates skin wound healing by promoting M2 macrophage polarization via suppression of Socs1
Source: Front Physiol. 2025 Oct 14;16:1638356. doi: 10.3389/fphys.2025.1638356 (PMC12559803; doi:10.3389/fphys.2025.1638356)

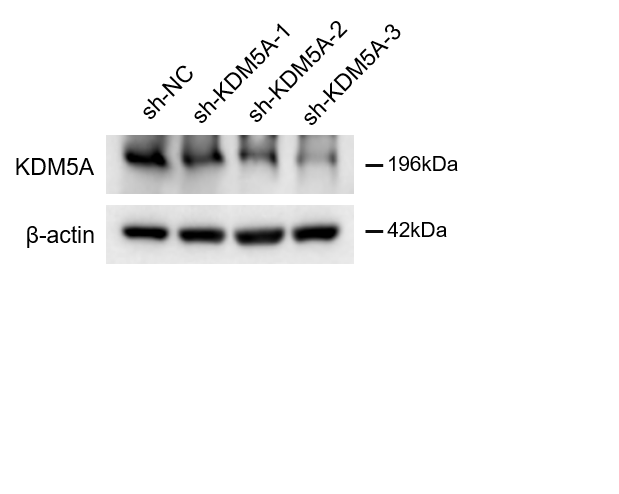


**Supplemental Fig.1** Knockdown of KDM5A was validated in NHDFs.

Supplement: Supplementary file 2 [file Supplementaryfile1.docx]
